# From mouse to man—a bridge too far?

**DOI:** 10.1093/nsr/nwz225

**Published:** 2020-01-06

**Authors:** Henry Kennedy, Colette Dehay

**Affiliations:** Univ Lyon, Université Claude Bernard Lyon 1, Inserm, Stem Cell and Brain Research Institute U1208, France; Institute of Neuroscience, Chinese Academy of Sciences, China; Univ Lyon, Université Claude Bernard Lyon 1, Inserm, Stem Cell and Brain Research Institute U1208, France

The contemplation of our demise, particularly via the loss of cognitive functions, is challenging for our species. While we seek to understand our brain and its action as a particularly intriguing manifestation of the physical universe, society also seeks solutions to concrete problems and, here, understanding the brain as an intellectual exercise must be extended to being able to maintain and repair its function. Just how difficult this is becomes apparent when one considers the biological functions of brains. The enormous capacity for adaptation of the mammal class is reflected by an extraordinary range of brain sizes from cortices of 8 kilograms to a mere fraction of a gram in certain rodents. Changes in brain size directly impact on the architecture of the cortex [1]. This poses an insoluble problem; a reductionist approach would lead us to assume that different sorts of mammalian brains are all doing the same thing, but the biological adaptation that they reflect argues against this notion [2].

Thirty years ago, the model of choice for studying neuronal mechanisms underlying cognitive function was the visual system of the awake-behaving monkey. This allowed recoding of single neurons and the investigation of the feedforward pathway leading to a deep understanding of the representation of the world via the construction of receptive fields across the cortical hierarchy. However, the advent of genetic tools in the mouse and the ability of optic control of neuronal activity has ensured that, in 2020, the mouse has largely replaced the macaque as the model of choice. But then we must wonder how relevant the mouse brain is for understanding the human brain.

Rodent models exploiting mouse transgenics have made brilliant progress in elucidating the cellular specifics of diverse aspects of cortical cellular mechanisms involved in motor and cognitive processes (e.g. [3,4]). Can we approach a similar level of understanding in the non-human primate cortex? Many cortical features are constant across species. This is exemplified by the preservation of the areal layout reflecting genetic regulation of conserved graded transcription pattern expression during corticogenesis [5] and many aspects of the specification, migration and cell-type differentiation to form functional circuits proceed in a stereotypical fashion across species [6]. However, key aspects of human perception, cognition and behavior appear to depend on primate-specific particularities of the cerebral cortex, and this may explain why so many human neurological and neuropsychiatric diseases are inadequately modeled in rodents [7]. Primate-specific specializations, we speculate, stem from the unique developmental processes found in human and non-human primates [8–10]. These specializations generate numerous *qualitative* differences between rodent and primate cortices, of which we cite five examples. (i) There are dramatic changes in the dimensions of pyramidal dendritic arbors across the cortex in primates [11, 12] that are theorized [13] to shape the observed hierarchy of timescales [14]); these gradients in pyramidal cell dimensions are absent in rodents [15]. (ii) The mouse anterior cingulate cortex has significant output to area V1 that is known to drive experience-dependent spatial and motor [16] expectations as well as spatial attention [17]. There are no such connections in primates, meaning that the anterior cingulate cortex and area V1 are not homologous in primates and rodents. (iii) There is a dense inter-areal network linking the primary sensory and motor areas in the mouse [18], providing the anatomical substrate for a rodent-specific multimodal integration [18–20]; this network is absent in the macaque [21]. (iv) A dramatic shift in cortical layer-specific gene expression is observed, from the infragranular layers in rodents to the supragranular layers in primates, that constitutes further evidence of the expansion and changing role of the upper layers in primates [22]. (v) Finally, recent claims in rodents of non-canonical cortico-cortical feedback loops [23] and non-canonical cortical microcircuits [24] suggest further evidence of circuit-level species differences.

Presently, researchers are partly overcoming the gap between mouse and primate by creating non-human primate (NHP) models of disease [25]. This research, largely led by China, goes towards solving the problem of a model organism for understanding pathology in so far as it allows invasive investigation of a genetically modified macaque. But is this going to allow sufficient progress in understanding the healthy primate brain? It could be argued that, until we know the neural mechanisms underlying perceptual and cognitive processes in the normal healthy brain are, we are not going to fully understand the pathologies of the cardinal features of human intelligence. The emerging model for understanding cortical processing is Bayesian inference that plays out in hierarchical cortical processing known as predictive coding, which directly concerns the integration of ascending, feedforward and descending feedback signals into the local circuit [26,27]. Predictive coding has been described as a strange inversion in which the brain, instead of being thought of as a stimulus–response link, is conceived as ensuring an inference providing explanations of our sensorium [28]. Feedforward pathways are thought to directly contribute to the elaboration of the receptive field from sensory input and, here, 50 years in electrophysiological experiments, first in cats, then monkeys and more recently in mice, have allowed a very detailed understanding. Predictive coding postulates that the feedback pathway relays predictions and here it has been much harder to determine the underlying neural mechanisms involved. Importantly, these feedback pathways are implicated in human neurological disorders including schizophrenia and autism [29]. We have shown that, in primates, there are multiple routes taken by feedback connections in the primate [30] and one can speculate that they will differently impact on the numerous functions subscribed to the feedback pathway including those proposed by predictive coding theory. Disentangling these diverse feedback pathways and determining the function of the circuits they support will crucially require the use of optogenetic tools. Could this work simply be carried out in the mouse? In addition to the myriad rodent–primate differences alluded to above, there are many reasons why one might imagine that top-down processes are quantitatively and perhaps qualitatively different across species with different capacities of behavioral plasticity, such as observed in mice versus monkeys. There is therefore an ethical argument that demands that this research is undertaken in macaques. This would require the creation of monkey transgenic lines expressing Cre in restricted layers and cell types to allow targeted circuit manipulation.

The reductionist approach supports the notion of a model organism. In addition to philosophical objections to excessive reductionism, there are pitfalls in thinking of the brains of even closely related species as being necessarily equivalent. For example, SRGAP2 is a gene that is implicated in neocortical development and undergoes human-specific duplications. Recently, experiments in mice have suggested that, in humans, SRGAP2 induces neotenic spine maturation [31]. The maintenance of juvenile features in the adult brain (neoteny) endows physiological properties to the adult human, via unique dendritic spine features that are presumably not found in non-human primates, including apes. These findings suggest that we can expect clear limitations in the concept of an NHP model for understanding the totality of the integrative neurobiology of the human brain, and therefore point to the need for humanization procedures, allowing the function of a human gene of interest to be studied. Interestingly, the relevance of the neoteny hypothesis for understanding the human brain has been elegantly explored by the generation of transgenic monkeys carrying human copies of the MCPH1 [32], revealing improved short-term memory and reduced reaction times in delayed match-to-sample tasks. Overall, these findings point to the need for a complex triangulation with investigation of different models but with a clear emphasis on the non-human primate. They also urge us to address the ethical issues raised by the nature of these experiments on our near cousins, as well as the ethics of not doing these experiments.
